# A designathon to collaboratively develop sustainable HIV prevention services for youth with community-based organizations in Nigeria

**DOI:** 10.1371/journal.pone.0322076

**Published:** 2026-07-29

**Authors:** Titilola Gbaja-Biamila, Oliver Ezechi, Ebenezer Adeoti, Jane Okwuzu, Chiyere Arinze, Lauren Fidelak, Olufunto Olusanya, Ucheoma Nwaozuru, Akeem Lateef, Temitope Ojo, Hong Xian, Tomilola Musari-martins, Abideen Salako, Agatha Wapmuk, Folahanmi Akinsolu, Suzanne Day, Nora Rosenberg, Aisha Koledowo, Jason Ong, Weiming Tang, Susan Nkengasong, David Oladele, Adesola Musa, Conserve Donaldson, Ross Brownson, Collins Airhihenbuwa, Joseph Tucker, Juliet Iwelunmor

**Affiliations:** 1 Division of Infectious Diseases, Washington University School of Medicine, Saint Louis, Missouri, United States of America; 2 Clinical Sciences Department, Nigerian Institute of Medical Research, Lagos, Nigeria; 3 Northwestern Medicine, Bluhm Cardiovascular Institute, Chicago, Illinois, United States of America; 4 Department of Medicine, Division of Infectious Diseases, University of North Carolina at Chapel Hill, Chapel Hill, North Carolina, United States of America; 5 Department of Implementation Science, Division of Public Health Sciences, Wake Forest School of Medicine, Winston-Salem, North Carolina, United States of America; 6 College for Public Health and Social Justice, Saint Louis University, Saint Louis, Missouri, United States of America; 7 Gillings School of Global Public Health, University of North Carolina at Chapel Hill, Chapel Hill, North Carolina, United States of America; 8 Department of Public Health, Lead City University, Ibadan, Oyo, Nigeria; 9 Faculty of Infectious and Tropical Diseases, London School of Hygiene and Tropical Medicine, London, United Kingdom; 10 The University of North Carolina at Chapel Hill Project-China, Guangzhou, China; 11 Faculty of Medicine, School of Translational Medicine, Nursing and Health Sciences, Monash University, Melbourne, Australia; 12 The George Washington University Milken Institute School of Public Health, Washington, District of Columbia, United States of America; 13 Prevention Research Center in St. Louis, Brown School at Washington University in St. Louis, St. Louis, Missouri, United States of America; 14 Department of Surgery (Division of Public Health Sciences) and Alvin J. Siteman Cancer Center, Washington University School of Medicine, Washington University in St. Louis, St. Louis, Missouri, United States of America; 15 Department of Health Management and Policy, School of Public Health, Georgia State University, Atlanta, Georgia, United States of America; SKYDA Health Nigeria, NIGERIA

## Abstract

**Introduction:**

The engagement of youth in the design of services that promote and increase the uptake of essential services to reduce HIV and other sexually transmitted infections is vital to sustaining these preventive services. The study objective is to identify strategies for sustaining HIV prevention services among Nigerian youth, in partnership with community-based organizations, through a crowdsourced open call and designathon in Nigeria.

**Materials and methods:**

From February to March 2024, Nigerian youth (ages 14–24) submitted ideas to an open crowdsourcing call on how community-based organizations in Nigeria might sustain HIV self-testing and youth-friendly preventive services for at-risk youth. The submissions were scored in each domain: relevance, novelty, scalability, replicability, potential for sustainability, and promotion of equity and fairness, with an integer score of 1 (low) to 3 (high)(S1). Ten teams were selected to participate in the 72-hour designathon. Both quantitative and qualitative analysis was conducted. The study used thematic analysis and the PEN 3-Cultural Model to categorize qualitative data into six themes. Descriptive statistics were used to calculate participants’ demographic characteristics.

**Results:**

One hundred and seventy-eight participants submitted entries; 161 were online entries, and 17 were received via WhatsApp. The majority of participants were female (57.1%) and aged 19–23 years (55.0%), with a mean age of 21.7 (±4.1). Most participants were from the southwest of Nigeria (53.8%), had secondary education (68.9%), and were students (67.9%). About one-third of teams (31.1%) indicated collaborating with non-governmental organizations as a promising way to sustain preventive services. Six themes emerged: Adapted Intervention Design and Delivery, Youth Engagement and Education, Organization Setting, Socio-cultural and Community Context, Community Leadership Through Training and Financial Resources, and Sustainment Through Community Collaboration.

**Conclusion:**

The crowdsourcing contest open call and designathon engaged youth from diverse backgrounds, and it is a practical way to generate solutions from key stakeholders affected by the community’s prevailing health issues. Developing service delivery strategies that engage, educate, and collaborate with youth on HIV prevention could be sustainable. However, this will not be conclusive without implementing them over time to test their feasibility and sustainability.

## Introduction

Sustaining youth-friendly preventive services is a critical challenge in Africa, particularly in Nigeria. Nigeria faces one of the highest burdens of HIV among youth in West and Central Africa. [1]. We will refer to youth in this paper as individuals aged 14–24. Despite the progress made in HIV prevention, there is still suboptimal uptake of HIV self-testing (HIVST) and Pre-Exposure Prophylaxis(PrEP) among Nigerian youth [2–4]. In addition, there are limited youth-friendly clinics that tailor services for youth [5]. This may be a result of societal stigma surrounding youth access to sexual and reproductive health services [6]. As well as the designs and processes within the existing youth-friendly centers, making them unpopular among the youth [6]. A key contributory factor to this gap is the fact that there is limited youth engagement in the design and implementation of these preventive services, which has led to the creation of interventions that are often misaligned with the needs of youth [7–9]. Engaging youth in creating and planning HIV prevention services is essential to boosting their uptake. Youth engagement in the design of health services has been observed to play a critical role in improving the uptake of preventive measures against HIV and other sexually transmitted infections (STIs) [10–13]. Creative approaches are needed to develop youth-engaged HIV prevention services in LMICs, particularly in Africa [14]. Crowdsourcing represents one way to enhance youth engagement in HIV prevention services.

### Crowdsourcing

Crowdsourcing involves having a group of people solve a problem and then sharing the solution with the public [15,16]. Crowdsourcing provides a structured, practical approach to enhance youth engagement in HIV research compared to youth advisory boards or similar conventional approaches [17,18]. Recently, studies have used crowdsourcing contest open calls to gather ideas and strategies from youth, leveraging their creativity and firsthand experiences to improve health services [19]. In Nigeria, the 4YouthByYouth initiative [20,21] has adopted this approach to solicit youth-driven ideas on how community-based organizations (CBOs) can sustain HIV self-testing and preventive services for at-risk youth. This method of participation empowers youth, encouraging them to contribute directly to creating more relevant and accessible health services, thereby nurturing a sense of ownership and increasing the likelihood of sustained engagement [22,23].

### Community-based organizations

Community-based organizations (CBOs) are vital in sustaining local health services by addressing their communities’ unique needs [24,25]. Community-based organizations (CBOs) develop tailored health interventions that are culturally relevant, accessible, and responsive to local challenges. They build community trust, encourage essential health services, and leverage local knowledge for better health outcomes [26]. CBOs nurture partnerships with government and donor organizations, obtaining funding for training and technical support [27]. They link communities and healthcare systems, mobilizing resources and engaging volunteers [28]. Therefore, using the PEN 3-Cultural Model in this study can elicit the youth’s perception of strategies that could lead to sustainable HIV prevention services with CBOs in the community.

### PEN-3 model

**Fig 1:**
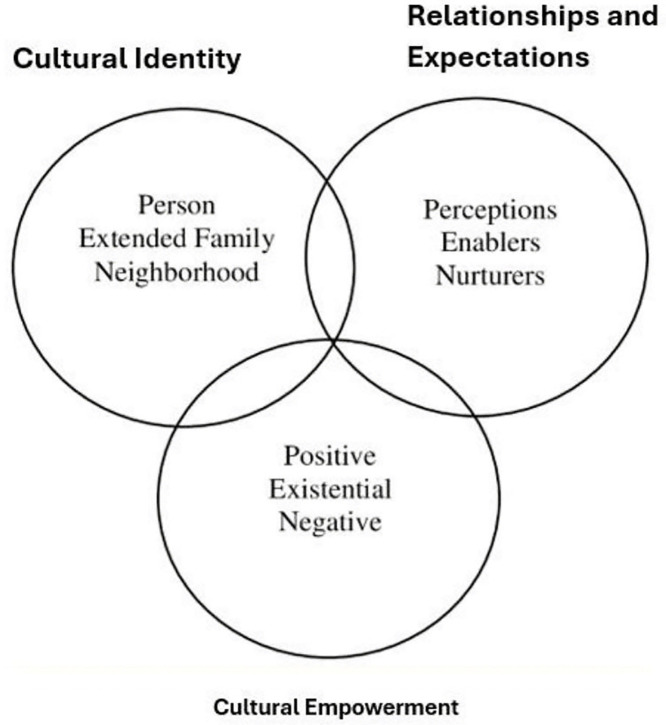
The adapted PEN-3 cultural model [31].

The PEN-3 Model (Fig 1) is a cultural model that emphasizes culture, health beliefs, behaviors, and health outcomes [29]. The PEN 3 model places culture at the center of effective public health intervention while addressing the development, implementation, and evaluation processes [29–31]. It focuses on how individuals’ perceptions and actions shape health beliefs and outcomes [29,30,32].

The PEN-3 Model aligns with activities and roles within CBOs. The domain of Cultural Identity – Neighborhood is an aspect where Community-Based Organizations (CBOs) are rooted, primarily located in local neighborhoods, and serve as trusted, relevant messengers and implementers in these communities [33]. The domain in the Model that highlights the Relationships and Expectations – Enablers and Nurturers align with CBOs’ role as enablers and nurturers, reinforcing health-promoting behaviors in communities. [34,35].

### Objective

The objective is to identify strategies to sustain HIV prevention services among Nigerian youth, in partnership with community-based organizations, through a crowdsourced designathon in Nigeria.

## Materials and methods

### Study design and crowdsourcing open call description

#### Study design.

This is a descriptive mixed-methods study.

#### Crowdsourcing open call.

It is a crowdsourcing open call that was conducted among youth in Lagos, Nigeria, in 2024. A multi-sectoral open-call advisory council comprising ten stakeholders from the government, media, research institutes, and youth organizations coordinated the crowdsourcing competition. This team was responsible for promoting, judging, and administering open calls.

#### Promotion and submission.

Submissions were solicited over seven weeks from February to March 2024 through various channels, including in-person encounters with 4YBY ambassadors, social media platforms (WhatsApp, Instagram, Facebook), print media, blogs, banners, e-flyers, and the 4YBY website. Potential participants were provided with instructions on submitting an entry via the open-call website. Team leaders filled in the forms where the data was collected. The data were recorded as a categorical variable indicating whether the participant responded via email, WhatsApp, or a Google form.

#### Eligibility criteria.

To be eligible to enter the open call, participants needed to meet the following eligibility criteria: they had to be between 14 and 24 years old, reside in one of the 36 states in Nigeria, be affiliated with a certified community-based organization that serves young people or addresses youth health, and form a team of 4 people.

Although teams made submissions, demographic data were captured only for those who submitted to their team (“Table 1”).

**Table 1:** Demographic information of participants who applied for the open call in March 2024.

Variables of all participants	N = 178 N (%)
**Gender**	
Female	108 (57.1)
Male	70 (42.9)
**Mean Age (SD)**	21.7 (±4.1)
**Age of Participants**	
14 - 18 years	29 (16.3)
19–23 years	104 (58.4)
24 and above	45 (25.3)
**Variables for teams eligible for judging after screening**	**N = 106**
**Geopolitical Zones for Teams**	
South-south	9 (8.5)
South-east	9 (8.5)
South-west	57 (53.8)
North-central	21 (19.8)
North-east	5 (4.7)
North-west	4 (3.8)
Missing (1)	
**The highest level of education for teams**	
Bachelors	33 (31.1)
Senior Secondary school	73 (68.9)
**Employment status for teams**	
Employed	22(20.8)
Unemployed	84 (79.2)
**Occupation**	
Professional/technical/Managerial	16 (15.1)
Student	67 (63.2)
Technical/skilled Manual	20 (18.9)
Unskilled Manual	3 (2.8)
**Heard of HIV Self-testing**	
No	8 (7.5)
Yes	97 (91.5)
I don’t know	1 (1.0)
**Heard of Pre-Exposure Prophylaxis**	
No	23 (21.7)
Yes	81 (76.4)
I don’t know	2 (1.9)
**Heard of the at-risk population for HIV infection**	
No	6 (5.7)
Yes	98 (92.5)
I don’t know	2 (1.8)
**Heard of the community-based Organizations**	
No	14 (13.2)
Yes	88 (83.0)
I don’t know	4 (3.8)
**Focal Areas of Community-based Organizations (CBOs) Collaborating with Teams**	
Agricultural	1 (0.9)
Community safety	9 (8.5)
Women	3 (2.8)
Health	2 (1.9)
Humanitarian Aid	6 (5.7)
Non-Governmental Organization	33 (31.1)
Unclassified	30 (28.3)
Religious	4 (3.8)
School	12 (11.3)
Youth	6 (5.7)

#### Scoring and selection.

At the end of the submission period, two research team members pre-screened all submissions to ensure they met eligibility criteria. Eligible submissions were then rated by three judges who independently reviewed each eligible submission. Using the four evaluation domains: Relevance, Novelty, Scalability/Replicability, Sustainability, and Promotion of Equity and Fairness, with an integer score of 1 (low) to 3 (high). These scoring domains were used based on previous designathons, which the team had conducted [12,36–41]. Relevance refers to whether the idea is relevant to Youth in the Nigerian context. Was the idea clearly described to showcase its relevance for AYAs in Nigerian communities? Novelty refers to the extent to which the submission was unique, innovative, and novel. In contrast, feasibility, Scalability, Replicability, and Sustainability refer to how easily and practically implementable the innovation would be in diverse Nigerian settings. Would it work in rural and/or urban settings in Nigeria? If there were discrepancies in scores, the judges would discuss until a consensus was reached, usually by assessing how each response was relevant to each scoring domain.

At the end of this judging process, the top 10 teams with the highest scores (>8) (S3 Table) were invited to the 72-hour designathon held between April 3–5, 2024, in Lagos.

#### Designathon process.

A designathon is a three-stage participatory activity informed by design thinking that includes preparation with end-users, intensive collaboration, and follow-up [42,43]. The designathon had ten teams design strategies to sustain HIV self-testing and youth-friendly preventive services for at-risk AYAs through CBO collaboration.

#### Sampling method.

This study used purposive Sampling to recruit participants. This method was used because our target group was youth who met the eligibility criteria previously described.

### Data collection

The crowdsourced open call solicited written ideas on the question “How community-based organizations in Nigeria might sustain HIV self-testing (HIVST) and youth-friendly preventive services for at-risk youth.” Prospective participants were asked to submit entries to respond to the open-call question. Four variables were gathered for every submission: the age, gender, and educational level of the individual submitting to the open call. The data were recorded as a categorical variable indicating whether the participant responded via email, WhatsApp, or a Google form. Google submissions were submitted on structured forms, whereas email and WhatsApp submissions were submitted without a form. All fields were required on Google Forms before submission.

During the final presentation of implementation strategies from the ten final teams in the designathon, four independent scoring criteria were utilized in the scoring process: (1) relevance, (2) novelty, (3) scalability/replicability, (4)sustainability, and (5) promotion of equity and Fairness. Scoring was conducted in April 2024, where each submission was assigned a score of 1–3 for each criterion (Relevance, Novelty, Scalability, Replicability, Sustainability, and Promotion of Equity and Fairness), with 3 being the highest score per criterion. The sum of the six judges’ integers was used as the total score, which ranged from 8 to 12. See S1 Table for a description of the scoring guide and the teams’ total scores. The participant’s open call entries were de-identified and transcribed. The entries submitted by the top ten teams that participated in the designathon were used as qualitative data for the study, and Table 1 presents the quantitative data derived from these submissions.

### Data analysis

Quantitative data analysis: A unique identifier was used to merge the scoring datasets into a single dataset across all four scoring criteria. Next, the entire dataset was cleaned to eliminate ineligible, vacant, and redundant submissions. The cleaning process included removing duplicates and missing data, identifying and correcting errors, and resolving inconsistencies. Additionally, we ensured that variable definitions were consistent across all data sources. All data cleaning and analysis were performed in SPSS version 29. Descriptive statistics were computed. Means and standard deviations were calculated for continuous variables using a t-test. Based on self-report, gender was dichotomized into male and female. Age was collected as a numerical value and categorized into 5-year intervals (14–18, 19–23, 24-, and above). Education level was a categorical variable based on the highest level of schooling completed (Bachelor, Senior Secondary school (equivalent to High school)).

The qualitative data consisted of the submissions to the open call. The six steps of Braun and Clarke’s thematic analysis were used to systematically analyze and code participants’ qualitative data to identify patterns and themes [44,45]. Two authors manually performed inductive thematic analysis, developing codes informed by the PEN-3 domains. Additionally, the PEN-3 model was utilized to assess the study’s qualitative data and capture the range of participants’ responses. The authors first thoroughly assessed the open call responses to become familiar with the entries and then generated initial codes by systematically labeling interesting or significant features of the data with codes that capture the core message of the open call. That is, creating the codes directly from the data itself in a “bottom-up” approach, using the PEN-3 domains. The codes were then sorted into potential themes and sub-themes by grouping them based on emerging patterns in the data. After this process, the themes were reviewed to assess whether they accurately reflect the participants’ views captured in the data. During this process, the authors combined, split, or discarded themes. Next, each theme was given a descriptive name and defined to explain how it helps understand the open call responses. Once these steps were completed, the final write-up was done. The PEN-3 cultural model (Fig 1) comprises three domains, with each domain consisting of three constructs that form the acronym PEN: (1) Cultural Identity domain (Person, Extended Family, Neighborhood), (2) Relationships and Expectations (perceptions, Enablers, and Nurturers), and (3) Cultural Empowerment (positive, Existential, and Negative) [31]The PEN-3 cultural model provided a conceptual framework for examining the data’s dimensions of cultural identity domain (Person, Extended Family, Neighborhood) and Relationships and Expectations (Perceptions, Enablers, Nurturers) [46,47]. The following six themes were identified: Adapted Intervention Design and Delivery, Youth Engagement and Education, Organization Setting, Socio-Cultural and Community Context, Community Leadership Through Training and Financial Resources, and Sustainment Through Community Collaboration. The trustworthiness of our analysis was ensured through the application of credibility, dependability, and confirmability. Credibility was enhanced through peer debriefing and fully comprehending the data. Dependability was achieved through detailed record-keeping and external audits. Confirmability was ensured through the researchers’ reflexive practices and direct quotes from participants.

### Ethical approval

The Nigerian Institute of Medical Research Institutional Review Board (Project number for approval: IRB/23/064) provided ethical approval to conduct the research. Due to the non-sensitive nature of the data, informed consent and assent were not required.

#### Inclusivity in global research.

Additional information on the ethical, cultural, and scientific considerations specific to inclusivity in global research is provided in Supporting Information S5 File.

## Results

### Descriptive analysis

**Fig 2:**
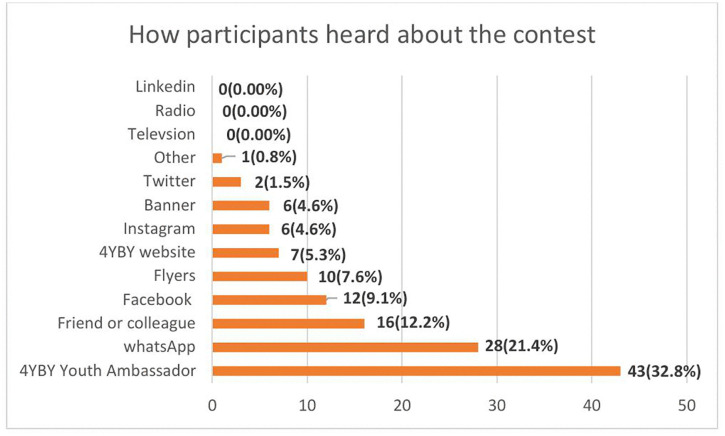
How participants heard about the context.

A total of 178 participants responded to the open call; most were female (57.1%) and aged 19–23 (55.0%). Data from the team leads (Table 1: Variables for team groups) showed that 17 had most of their data missing; these were sent via WhatsApp and so were excluded from the analysis (most of their demographic data was missing); among those whose data was used in the study, the majority were from the southwest (53.8%), had completed secondary school (68.9%), and were students (63.2%). Most of the participants had heard of HIV self-testing (91.5%), had heard of PrEP (76.4%), but this knowledge did not assess the various levels of expertise, and had heard of the at-risk population for HIV infection (92.5%). Most participants indicated they would work with non-governmental organizations (31.1%). After the screening process described in the materials and methods section, 106 participants participated in the designathon. Many participants heard about the open call through the 4YBY youth ambassadors (32.8%), as seen in Fig 2.

### Thematic analysis and the adoption of the PEN-3 cultural model (“Table 2”)

#### Adapted intervention design and delivery (perceptions-person).

All the teams had Pre-exposure prophylaxis (PrEP) and HIV self-testing as their interventions. This aligns with the quantitative data, which indicated that most of the youth had heard about PrEP and HIV self-testing, and this may be why all of them wanted these two interventions as part of their sustainment plan. Only a few had STI testing (#1, #5) as part of their intervention. However, Delivery modalities varied, including community outreach in partnership with youth groups and CBOs, mobile clinics, and digital platforms.

**Table 2:** Application of the PEN-3* cultural model on Ten teams strategy.

PEN-3 Domain – sub-domain	Theme	Illustrative Quote	Source
Perception-Person	Adapted Intervention Design and Delivery	*“Mobile Clinics:…..Schedule regular visits to communities, schools, and events, publicizing the clinic’s schedule through local channels and social media. Offer testing services free of charge or at subsidized rates to ensure affordability for youths.”*	#3
		*“Utilizes mobile outreach units equipped with educational materials, HIV self-testing kits, and STI testing supplies.”*	#4
		*“Leverages digital health solutions to improve access to sexual health services.”*	#2
		*“The Mobile Outreach for Youth Sexual Health Empowerment (MOY SHE) project has been initiated in collaboration with local community organizations (CBOs). MOY SHE employs a comprehensive approach to reach out to young individuals where they are by utilizing mobile outreach units.”*	#5
		*“Outreach events can effectively engage at-risk youth and foster a sense of belonging and trust, utilizing interactive and youth-friendly methods such as games, role-plays, and multimedia resources.”*	#10
Enabler -Person	Youth Engagement and Education	*“The first step involves training committed CBO members in these programs, who act as peer educators and assist in disseminating information.”*	#1
		*“Engaging Students and Young People: School Clubs and SHR Ambassadors: Establish school clubs and train students to become peer educators and ambassadors for sexual and reproductive health (SHR) in their schools Peer education networks: Leverage trained youth ambassadors to spread awareness through their social circles.”*	#2
		*“Engaging Students and Young People: School Clubs and SHR Ambassadors: Establish school clubs and train students to become peer educators and ambassadors for sexual and reproductive health (SHR) in their schools Peer education networks: Leverage trained youth ambassadors to spread awareness through their social circles.”*	#2
		*“Encourage peer advocacy by appointing ambassadors among our community-based members, equipping them with knowledge and skills to facilitate the awareness and project simulations of HIV preventive services.”*	#4
Enabler – Neighborhood	Socio-cultural and community context*	*“ Culturally competent training: Training materials would be customized to the target population’s unique needs, local language, and cultural quirks. This promotes greater comprehension and raises the program’s communal relevance.”*	#1
		*“Ensuring at-risk youths have continual access to vital information about HIV and STIs: Partnering with local artists to create informative street art and murals in areas frequented by youths.”*	#7
		*“…we strive to deliver comprehensive sex education in a culturally sensitive and engaging manner.”*	#8,#9
	Organization setting	*“Partnerships with Schools…… Integrate HIV education and prevention services into school curricula and activities. Action Plan: Collaborate with education authorities to develop age-appropriate HIV curriculum materials for schools.”*	#3
		*“Inclusive Youth Space: As an organization, we emphasize creating inclusive youth spaces for effective HIV preventive services. These spaces ensure services are tailored to the specific needs of young people, fostering a comfortable and safe environment.”*	#9
		*“Train-the-trainer model: A dedicated group of CBO members will receive extensive instruction on PrEP, STI testing, and HIV self-testing. After that, these people will serve as peer educators.”*	#1
		*“Peer-to-Peer Training: Sensitization and Information Sharing: Train young people to sensitize their peers about HIV self-testing, preventive services, and responsible sexual behavior.Reaching Diverse Groups: Conduct training sessions in different locations to cater to various communities and groups.”*	#2
		*“ Financial support: Advocate for increased funding and resource allocation for youth-focused HIV prevention programs.”*	#2
	Community Leadership Through Training and Financial Resources	*“Peer Education Programs: Train youth to educate their peers about HIV prevention methods, including self-testing.”*	#3,#1
		*“Peer Navigators: Young people are more than willing to participate in program development and management. ”*	#6
		*“While some teams would use “Leveraging platforms like Facebook, Instagram, and Twitter, ……..will be utilized to make information dissemination engaging.”*	
		*“Organize peer-led workshops, seminars, and discussions in schools, communities, and online platforms.”*	#3 #9
		*“Plan for sustainability by judicious management of finance and resources, merging free availability to the less privileged population, minimal subsidy to the average socioeconomic population, and market value rate to the high-profile population.”*	#4
Nurturer – Neighborhood	Sustainment Through Community Collaboration	*“Partnerships with Schools…… Integrate HIV education and prevention services into school curricula and activities. Action Plan: Collaborate with education authorities to develop age-appropriate HIV curriculum materials for schools.”*	#3
		*“Collaboration: The CBOs are linked up with youth centers closest to them, where they communicate with members of the communities. A person who wants a follow-up would know who to reach out to and how to move forward.”*	#1
		*“Harness a wide range of partnerships with community stakeholders, health facilities, organizations, and individuals (inclusive of members of our community-based organization), to facilitate accessibility to the target population, for referrals and usage of available clinical assistance to vulnerable individuals, for financial & resource aid, and for sustainability.”*	#4

The Extended Family had no quotes or themes, so it was not included in the table.

*“Mobile Clinics:…..Schedule regular visits to communities, schools, and events, publicizing the clinic’s schedule through local channels and social media. Offer testing services free of charge or at subsidized rates to ensure affordability for youths.” (#3)*

*“Develop a mobile app or website dedicated to HIV prevention, self-testing.”(#3)*

*“Outreach events can effectively engage at-risk youth and foster a sense of belonging and trust, utilizing interactive and youth-friendly methods such as games, role-plays, and multimedia resources.” (#10)*

#### Youth engagement and education (enablers-person).

The teams’ projects involved engaging the community “*Community Sensitization and Awareness Raising (#2)* “ and dialogue, as well as ensuring the use of local resources to promote community ownership of their strategies, which was targeted to uniting the youth and CBOs in a way that would increase the sustainability of the project:

*“Engaging Students and Young People: School Clubs and SHR Ambassadors: Establish school clubs and train students to become peer educators and ambassadors for sexual and reproductive health (SHR) in their schools. (#2)*

*“Entertainment: Community youth engagement through entertainment education can also be very effective in increasing HIV self-testing and other preventive services.” (#6)*

*“Sports Tournaments: Organizing football, basketball, or volleyball tournaments in local communities and urban areas, providing opportunities for youth to showcase skills and compete for prizes.” (#7)*

#### Organization setting (enablers-neighborhood).

The teams were all engaged with CBOs, but these organizations differed in objectives and the area they worked in. This aligns with the quantitative analysis, which showed that the CBOs had different focus areas, and this is reflected in the quantitative analysis. Teams have different CBOs affiliated with them and therefore have different strategies for sustaining youth-friendly services; some teams would engage with schools, while others felt that organizational settings should have safe spaces for youth. While some teams felt the inclusion of a safe space for youth would foster better engagement for youth in an organization setting for preventive services,

*“Partnerships with Schools…… Integrate HIV education and prevention services into school curricula and activities.”* (#3)

*“Inclusive Youth Space: As an organization, we emphasize creating inclusive youth spaces for effective HIV preventive services. These spaces ensure services are tailored to the specific needs of young people, fostering a comfortable and safe environment.”* (#9)

### Socio-cultural and community context (enablers-neighborhood)

Cultural considerations were incorporated into some of the team’s projects to ensure information delivery was more acceptable and relevant to the community. Some teams felt this would increase the understanding of HIV prevention as well as improve the uptake of the services they were creating:

*“Culturally competent training: Training materials would be customized to the target population’s unique needs, local language, and cultural quirks.” (#1)*

*“Ensuring at-risk youths have continual access to vital information about HIV and STIs: Partnering with local artists to create informative street art and murals in areas frequented by youths.” (#7)*

*“…we strive to deliver comprehensive sex education in a culturally sensitive and engaging manner.” (#8.#9)*

### Community leadership through training and financial resources (enablers-neighborhood)

All teams emphasized training, particularly peer-to-peer education and digital information sharing, as key strategies for sustainability**: *“*** *Peer Education Programs: Train youth to educate their peers about HIV prevention methods, including self-testing (#3,#1)”* while some teams would use digital platforms to disseminate their information ***“****Leveraging platforms like Facebook, Instagram, and Twitter, ……..will be utilized to make information dissemination engaging (#9)****”***, just three teams mentioned financial support as a key point to address these aspects were part of their sustainment plan to sustain HIV self-testing (HIVST) and youth-friendly preventive services:

*“Financial support: Advocate for increased funding and resource allocation for youth-focused HIV prevention programs(#2)”*,

*“Train-the-trainer model: A dedicated group of CBO members will receive extensive instruction on PrEP, STI testing, and HIV self-testing. After that, these people will serve as peer educators. (#1)”*

*“Peer-to-Peer Training: Sensitization and Information Sharing: Train young people to sensitize their peers about HIV self-testing, preventive services, and responsible sexual behavior.” (#2)*

#### Sustainment through community collaboration (nurtures-neighborhood).

Teams’ projects highlighted the creation of champions and, with all the teams, the collaboration of local CBOs within the community; the team’s perceptions were that it was key to involve stakeholders in the community, not just the youth, to enable the sustainment of their project:

***“*** *Organize peer-led workshops, seminars, and discussions in schools, communities, and online platforms.” (#3)*

***“*** *Collaboration: The CBOs are linked up with youth centers closest to them, where they communicate with members of the communities. A person who wants a follow-up would know who to reach out to and how to move forward.” (#1)*

*“Harness a wide range of partnerships with community stakeholders, health facilities, organizations, and individuals (inclusive of members of our community-based organization) to facilitate accessibility to the target population, for referrals and usage of available clinical assistance to vulnerable individuals, for financial & resource aid, and for sustainability.” (#4)*

## Discussion

The findings of this study highlighted the potential of CBOs to sustain HIV prevention services among youth through innovative strategies obtained during the crowdsourcing open call and designathon. Most youth collaborated with non-governmental, community-based organizations they believed would sustain HIV prevention services. The majority of youth perspectives on strategies they identified for collaborating with CBOs to sustain HIV preventive services focused on adapting intervention design and delivery of HIV services, youth engagement, education, and community collaborations with various stakeholders and institutions.

This study offers several innovative contributions to HIV prevention among youth. First, it demonstrates the unique potential of CBOs to collaborate directly with youth in designing strategies for sustaining HIV prevention services, an approach that contrasts with previous top-down interventions which have been implemented such as Integration in Primary Healthcare [48], Public‐private partnerships [49], integrating HIV/AIDS services into Universal Health Coverage [49] and. Integrating HIV/AIDS services into financial protection systems [50]. By adopting a bottom-up, youth-driven designathon, this work provides new evidence that community and youth ownership can strengthen sustainability by enhancing trust, cultural alignment, and the feasibility of implementation. Second, applying the PEN-3 model enabled us to identify cultural identity, particularly person- and neighborhood-level factors, that shaped youth-generated strategies for sustaining HIV services. Notably, while cultural empowerment was less frequently emphasized, this may reflect the inherent strength already embedded in youth–CBO partnerships, which serve as a form of community empowerment in their own right. Together, these findings advance current knowledge by illustrating how culturally grounded, youth-led, and CBO-supported approaches can yield HIV prevention strategies and interventions that are relevant and sustainable for Nigerian youth.

The youth-driven interventions highlighted the need to adapt designs and delivery processes to meet this population’s specific needs and preferences, which is similar to other studies in this area [51–53]. One of the key solutions found in this study, which is comparable to other studies with youth participants was the need for tailored HIV prevention services that leverage mobile clinics as avenues for community outreach; these services are accessible and can reach diverse youth, particularly in underserved or high-risk communities [54–57]. This approach can help overcome barriers such as stigma, transportation challenges, and time constraints [58]. Similarly, these barriers can occur in these clinics, including a lack of privacy, inadequate hours of operation, and poor tracking of patient referrals [59]. This method aligns with documented evidence of community-based HIV testing strategies as an effective means of identifying HIV at-risk youth who may not be aware of their HIV status, thereby offering these youth timely intervention, treatment and care [56]. The youth also indicated that integrating mobile clinics with digital health tools, such as telemedicine and mobile applications, may further enhance service sustainability, accessibility, and continuity of care [60–63]. Although this was the perception of the youth, some studies have found that in resource settings, the effectiveness of these strategies is hampered due to challenges, such as a lack of reliable internet and funding [64,65]

There was also an emphasis on engaging and educating youth, a critical component of effective HIV prevention services. Engagement and education of youth are vital for comprehensive HIV prevention programs that extend beyond the conventional clinical setting [66,67]. Peer education and youth-led advocacy initiatives can promote community knowledge dissemination, reduce misinformation, and nurture a supportive and sustainable environment for HIV prevention services among youth [68–70]. These activities will require collaborations with community-based organizations (CBOs). Community-based organizations can have different focus areas within the community, as seen in the CBOs with which the youth are collaborating in this study. For a program to be sustainable for years, it has to be cost-effective. Collaborations with CBOs can be cost-effective, particularly when considering wider reach in the community and leveraging existing community networks to gain insights from local perspectives [71,24]. Although collaborations with CBOs appear to be positive, they too face challenges, such as organizational and resource deficiencies and sociocultural barriers, which could affect their sustainability and community engagement activities [72–74]. It is, however, essential to note that the effectiveness of a sustainable project depends on the specific project, the type of collaboration, and careful management of resources and shared goals [24].

The sustainability of HIV prevention services requires strong partnerships with schools, churches, and other CBOs [66,67,72,73]. These institutions serve as trusted community anchors that can facilitate ongoing education, awareness, and service delivery. Schools can integrate comprehensive sexual health education into curricula, while faith-based organizations can provide culturally sensitive discussions on HIV prevention [74,75].

Utilizing the Crowdsourcing open call and designathon in this study provided an inclusive platform for youth to voice their needs and preferences. These activities can be conducted when sponsorship is available, provided by individuals or organizations. Crowdsourcing open call and designathon can be sustained through community engagement, collaboration, long-term Planning, and Strategic Alignment.

By actively involving youth in designing and implementing interventions, CBOs can ensure that services are relevant, culturally appropriate, and responsive to youth-specific concerns. Some limitations of this study were that the crowdsourcing open call and designathon were capital-intensive, which was mitigated by using local resources and volunteers who facilitated and served as mentors during the event. The judges in the open call and designathon did not have outcome data for selected proposals that had the potential to be sustainable, novel, scalable, replicable, and to promote equity and fairness. The 72 hrs. during which the designathon was held were not enough time for youth to develop a comprehensive, sustainable plan for youth HIV prevention services. However, the finalists of this designathon were invited to a four-week training bootcamp, where these ideas were further developed with the aim of implementation (S2 Table). The implication of this study, incorporating youth perspectives in collaboration with CBOs in HIV prevention strategies, is that it could promote more inclusiveness, enhance program effectiveness, reduce stigma, ensure youth-friendly services, and spur long-term sustainability.

## Conclusion

Different community-driven approaches to sustaining HIV prevention services among youth were obtained from the open call. This study is the first in Africa, using a ‘bottom-up approach’ to examine the sustainment of HIV prevention services for youth through direct collaboration with community-based organizations, while explicitly addressing the cultural influences shaping health behaviors. This represents a novel contribution to existing knowledge. Findings from this study will directly inform the development of a nationwide sustainment plan for youth-focused HIV services in Nigeria. This future research would explore scalable implementation strategies and evaluate the long-term impact of these community-based interventions on HIV prevention outcomes among youth.

## Supporting information

S1 Table Table of the designathon scoring guide description. (DOCX)

S2 Table Table of the top four teams at the designathon open call. (DOCX)

S3 Table Table of the team’s mean scores from the designathon open call. (DOCX)

S4 Table Table of demographic information of participants of the top 10 teams. (DOCX)

S5 File Inclusivity in global research. (DOCX)
