# Supplementary material for: A designathon to collaboratively develop sustainable HIV prevention services for youth with community-based organizations in Nigeria
Source: PLoS One. 2026 Jul 29;21(7):e0322076. doi: 10.1371/journal.pone.0322076 (PMC13419191; doi:10.1371/journal.pone.0322076)
Supplement: S1 Table — (DOCX) [file pone.0322076.s001.docx]

**Table shows the designathon scoring guide description.**

| **Total Score** | **Scoring Guide Description** |
| --- | --- |
| 4 | Submission meets none of the criteria (it is not clearly presented, not relevant, not innovative, not feasible, and has no potential for a positive impact on community-based organizations to sustain HIV testing and youth-friendly preventive services in Nigeria. |
| 5 – 6 | Submission meets some of the criteria but is weak. |
| 7 – 8 | Submission meets some of the criteria and has moderate strength. |
| 9 – 10 | Submission meets all of the criteria with moderate strength. |
| 11 – 12 | Submission meets all criteria, with exceptional strength (very clearly presented, highly relevant, extremely innovative, highly feasible, and has exceptional potential for positive impact on community-based organizations to sustain HIV testing and youth-friendly preventive services in Nigeria). |
