# Supplementary material for: A designathon to collaboratively develop sustainable HIV prevention services for youth with community-based organizations in Nigeria
Source: PLoS One. 2026 Jul 29;21(7):e0322076. doi: 10.1371/journal.pone.0322076 (PMC13419191; doi:10.1371/journal.pone.0322076)
Supplement: S2 Table — (DOCX) [file pone.0322076.s002.docx]

**The top four teams at the designathon open call.**

There was an even mix of a team from the southwest (Oyo state) and two from the north-central region of Nigeria. They all had similar approaches to sustaining youth-friendly preventive services, including peer education programs, mobile clinics, community outreach events, youth-friendly spaces to promote preventive services, and capacity building of youth and school partnerships. CBOs' targeted implementation strategies proposed by the second and third runners-up were similar.

Table showing characteristics of the Top three teams in the designathon

| **Rank** | **Team Characteristics** | **Members** | **State** | **Community-based organization (CBO)** | **Project Proposal Approaches** | **CBO targeted strategy** |
| --- | --- | --- | --- | --- | --- | --- |
| 1 | 22-24  Two Female  Two Males | 4 | Oyo | Slum and Rural Health Initiative (SRHIN) | Working with Peer Navigator to Engage young people in program development and management and leveraging peer networks.  • Educate and encourage peer screening, linking them to youth-friendly centers and schools and helping them access services.  • Share HIV and STI-related knowledge.  •Using Entertainment to engage young people in scriptwriting and role assignment in short films.  •Engaging the Community Youth-Friendly Centers in activities like friendly matches, picnics, and competitions to promote HIV services among young people. | 1. SRHIN’s goal is to increase public access to health professionals through advocacy (42), technology, and research so people can make the best health decisions possible. 2. they will empower one million youth by 2024 through volunteers, advocacy, and community partnership (42). |
| 2 | 20 -23 years  Three females, One male.  Students | 4 | Abuja | Education As A Vaccine (EVA) | The project aims to create a sustainable HIV self-testing model for at-risk Nigerian adolescents and young adults through school clubs, community sensitization, and confidential sharing. It includes peer-to-peer training, creative outreach, and capacity building, empowering young people to become health advocates and ensuring long-term sustainability. | 1. EVA improves the health and development of children, adolescents, and young people. 2. Community-based organizations are engaged through capacity building, financial support, and collaborative partnerships. This comprehensive approach empowers young people to become health advocates and ensures the long-term sustainability of HIV self-testing and preventive services. |
| 3 | 21 -23  All Male  Tertiary students | 4 | Oyo | Center For Youth Initiative on Self-Education | Community-based organizations (CBOs) can sustain HIV preventive programs by training dedicated members, improving awareness through community engagement, and building sustainability through social enterprise. This approach empowers communities to take control of their health, contributing to the long-term success of HIV prevention initiatives and ensuring affordability through discounted supplies and collaboration with healthcare professionals. | 1. Training of CBO peer educators, 2. Promoting awareness of HIV, PrEP, and STI through their local network and collaborating with local youth centers. 3. Building sustainability through social enterprise, integrating these services into existing programs through a remuneration system. By collaborating with distributors or healthcare professionals, |
| 3 | 22 -24 years  Three females, One male.  Students | 4 | Abuja | Aids healthcare foundation AHF | The text outlines several action plans for HIV prevention, including peer education programs, mobile clinics, community outreach events, youth-friendly spaces, digital platforms, capacity building, school partnerships, and monitoring and evaluation. | 1. AHF offers tailored interventions, trust, and culturally sensitive services, ensuring sustainability, maximizing HIV prevention impact, and promoting behavior change and improved treatment for HIV-positive individuals. 2. This will be done through peer education programs, mobile clinics, community outreach events, youth-friendly spaces, digital platforms, capacity building, school partnerships, and monitoring and evaluation. |
