# Supplementary material for: A designathon to collaboratively develop sustainable HIV prevention services for youth with community-based organizations in Nigeria
Source: PLoS One. 2026 Jul 29;21(7):e0322076. doi: 10.1371/journal.pone.0322076 (PMC13419191; doi:10.1371/journal.pone.0322076)
Supplement: S3 Table — (DOCX) [file pone.0322076.s003.docx]

Table showing the team's mean scores from the designathon open call

| Teams | Mean Relevance score (1-3) | Mean Novelty  Score (1-3) | Mean Feasibility, scalability/ replicability, and sustainability Score (1-3) | Mean Promotion of equity and fairness  Score (1-3) | Mean Teamwork Score  (1-3) | Mean Total Points |
| --- | --- | --- | --- | --- | --- | --- |
| ANTEC | 2.5 | 1.5 | 2 | 1.5 | 2 | 9.5 |
| BRIDGE | 2.5 | 1.5 | 2.5 | 2.5 | 2.5 | 11.5 |
| CHANGE | 1.5 | 1.5 | 1.5 | 2 | 2.5 | 9 |
| ELITE | 2.5 | 2 | 2.5 | 2 | 3 | 12 |
| GLOBAL IMPACT | 2 | 2 | 1.5 | 3 | 3 | 11.5 |
| GOAL GETTERS | 2 | 2 | 1.5 | 2 | 3 | 9.5 |
| INFINITY | 3 | 3 | 1.5 | 2 | 3 | 12.5 |
| PANACEA | 2.5 | 3 | 1.5 | 2 | 3 | 12 |
| PASA | 2 | 1.5 | 1.5 | 1.5 | 2 | 8.5 |
| EMPOWERMENT | 2.5 | 1 | 1 | 1.5 | 2.5 | 8.5 |
| ANTEC | 2.5 | 1.5 | 2 | 1.5 | 2 | 9.5 |

Team Infinity obtained the highest score.
