# Supplementary material for: A designathon to collaboratively develop sustainable HIV prevention services for youth with community-based organizations in Nigeria
Source: PLoS One. 2026 Jul 29;21(7):e0322076. doi: 10.1371/journal.pone.0322076 (PMC13419191; doi:10.1371/journal.pone.0322076)
Supplement: S4 Table — (DOCX) [file pone.0322076.s004.docx]

|  | **N= 36*n (%)** |
| --- | --- |
| **Gender**  Female  Male | 22 (61.1)  14 (38.9) |
| **Mean Age (SD)** | 22.3 (±1.39) |
| **Age of Participants**  19 - 21 years  22 – 24 years  Missing | 8 (22.2)  27 (75.0)  1 (2.8) |
| **States for teams**  Cross River  Ondo  Anambra  Lagos  Abuja  Ogun  Oyo | 4 (11.1)  3 (8.3)  4 (11.1)  4 (11.1)  8 (22.2)  2 (5.6)  11 (30.6) |
| **Highest level of education for teams**  Senior Secondary school  Bachelors  Postgraduate | 20 (55.6)  9 (25.0)  7 (19.4) |
| **Occupation**  Interns  National Service participant  Student | 2 (5.6)  3 (8.3)  31 (86.1) |

**S 4: Table of demographic information of participants of the top 10 teams**

(* four participants did not fill their demographic data during the designathon, so their data is missing)
